# Correlation of serum interleukin-6 levels and neutrophil-lymphocyte ratio in the severity of COVID-19

**DOI:** 10.12688/f1000research.132157.1

**Published:** 2023-09-22

**Authors:** Tenri Esa, Budu Budu, Budi Mulyono, Gita Vita Soraya, Andi Nilawati Usman, Umi Solekhah Intansari

**Affiliations:** 1 Faculty of Medicine, Hasanuddin University, Makassar, South Sulawesi, 90245, Indonesia; 2 Graduate School, Hasanuddin University, Makassar, South Sulawesi, 90245, Indonesia; 3 Faculty of Medicine, Public Health and Nursing, Gadjah Mada University, Yogyakarta, Special Region of Yogyakarta, Indonesia

**Keywords:** Coronavirus disease-19, COVID-19, Interleukin-6, IL-6, neutrophil–lymphocyte ratio, serum level, disease severity, inflammation

## Abstract

**Background:** Interleukin-6 (IL-6) is a pro-inflammatory cytokine that is produced at varying levels in patients with coronavirus disease 2019 (COVID-19). The neutrophil–lymphocyte ratio (NLR) is one of the new inflammatory markers of COVID-19. This study aimed to evaluate the differences in IL-6 level and the NLR in mild and severe COVID-19 and assess their correlation with COVID-19 severity and the correlation of IL-6 and NLR in COVID-19.

**Methods:** A total of 91 patients with COVID-19 were divided into mild (n = 57) and severe (n = 34) COVID-19 groups. IL-6 levels were measured using the electrochemiluminescence immunoassay method on Roche Cobas e411. The NLR was the ratio of the total neutrophil and lymphocyte counts from complete haematology on the Sysmex XS-800i. Data were analysed using the Kolmogorov–Smirnov, Mann–Whitney, receiver operating characteristic curve, chi-square and Spearman correlation tests. The statistical test was significant at p <0.05.

**Results:** Serum IL-6 levels and NLR significantly differed in mild and severe COVID-19. The median (min–max) IL-6 levels for mild and severe COVID-19 were 3.59 (1.50–638.30) pg/mL and 28.82 (5.52–926.30) pg/mL, respectively (p <0.001). The median (min–max) NLR in mild and moderate COVID-19 was 2.18 (0.69–15.58) and 8.13 (2.24–30.90), respectively (p <0.001). The obtained cut-off values for IL-6 and NLR were >6.99 pg/mL and >4.18, with odds ratios of 29.29 and 26.19, respectively. A positive correlation was found between IL-6 and NLR and COVID-19 severity (r = 0.612; p <0.001).

**Conclusions:** The results indicated that serum IL-6 levels and NLR are higher in severe COVID-19 than in mild COVID-19. Patients with IL-6 levels >6.99 pg/mL and NLR >4.18 are 29 and 26 times more likely to suffer from severe COVID-19, respectively. Serum IL-6 levels and NLR are strongly correlated with COVID-19 severity. Serum IL-6 levels correlate with NLR in COVID-19.

## Introduction

Coronavirus disease 2019 (COVID-19) is atypical pneumonia caused by the novel coronavirus of the coronaviridae class, i.e. severe acute respiratory syndrome coronavirus 2 (SARS-CoV-2).1 The World Health Organisation (WHO) declared this disease a pandemic. ^1^^,^^2^ SARS-CoV-2 can fatally lead to acute respiratory distress syndrome (ARDS) or multiple organ distress syndrome (MODS). ^1^^,^^3^

Based on WHO and the Indonesian Ministry of Health data for COVID-19 as of 19 August 2021, the number of confirmed cases globally reached 209,201,939, with a total of 4,390,467 deaths (case fatality rate [CFR] 2.1%). ^4^^,^^5^ In Indonesia, as of 19 August 2021, 3,930,300 confirmed cases, 3,472,915 recovered cases and 122,633 deaths from COVID-19 were reported (CFR 3.1%). South Sulawesi recorded 99,791 confirmed COVID-19 cases, 86,349 recovered cases and 1,807 deaths from COVID-19. ^4^

The main characteristic of people with COVID-19 is an increase in the production of pro-inflammatory cytokines. ^6^ Excessive increase in cytokines and inflammatory mediators such as interleukin (IL)-1β, IL-2, IL-6, IL-7, IL-8 and IL-10 and chemokines (CXCL 10 and CCL2) can exacerbate COVID-19 symptoms, which is characterised by a picture known as a ‘cytokine storm’. ^6^^,^^7^ In patients with COVID-19, high IL-6 levels were found as an immunological response to SARS-CoV-2. ^8^ Similarly, Coperchini *et al*. (2020) and Liu *et al*. (2020) demonstrated high serum IL-6 levels in patients with COVID-19 and circulating levels were positively related to disease severity. ^6^^,^^8^ Several studies have found high IL-6 levels in severe conditions or ARDS compared with mild cases; thus, IL-6 could be a biomarker of COVID-19 severity. ^9^^,^^10^

Luo *et al*. (2020) and Liu *et al*. (2020) stated that the combination of IL-6 levels >20 pg/mL and CD8+ T lymphocytes <165 cells/μL is a prognostic indicator and can predict COVID-19 death. ^11^^,^^12^ An increase in IL-6 levels of more than 10 times correlated with severe COVID-19, and decreased levels were related to successful therapy. ^11^^,^^12^

Studies have also found that the neutrophil–lymphocyte ratio (NLR) increased in COVID-19. This ratio is a new marker for assessing inflammation, including COVID-19. The NLR is obtained from a comparison of neutrophil and lymphocyte counts from a complete blood count. ^13^ A high NLR is caused by an imbalance in the expressions of inflammatory cytokines and the upregulation of genes involved in the apoptotic pathway of lymphocyte cells during SARS-CoV-2 infection. ^14^^,^^15^ A high NLR in patients with severe COVID-19 is characterised by lymphopenia and increased absolute neutrophil count; thus, the NLR may reflect an imbalance in inflammatory and immune responses in patients with COVID-19. ^16^

Ongoing research on IL-6 and NLR in COVID-19 reveals a correlation between IL-6 and NLR with COVID-19 severity. Several studies have also reported different cut-offs. However, currently, no study in Indonesia has reported cut-offs for IL-6 and NLR. The results of this study are expected to provide information regarding the body’s immune response and inflammation in COVID-19, which is characterised by differences in serum levels of the pro-inflammatory cytokine IL-6 and an imbalance of inflammatory cells with the NLR. The results can be included in COVID-19 management.

The hypothesis is:

1. IL-6 levels and NLR are higher in severe COVID-19 sufferers than mild ones.
2. IL-6 and NLR are positively correlated with the severity of COVID-19.
3. The higher the IL-6 and NLR cutoff, the more severe the symptoms of COVID-19.
4. IL-6 serum levels are associated with NLR in COVID-19 sufferers.

## Methods

### Population and research participants

The study population included individuals suspected of COVID-19 who underwent examinations and received treatment at the UNHAS Public Hospital and Makassar City Hospital, Makassar, South Sulawesi, Indonesia. The research participants were COVID-19 sufferers who met the inclusion and exclusion criteria. Patients with COVID-19 are those with COVID-19 confirmed by reverse-transcription polymerase chain reaction (RT-PCR) and diagnosed by a clinician at the Department of Internal Medicine/Pulmonary Medicine, Hasanuddin University, through anamnesis and physical, laboratory and radiological examinations. While the exclusion criteria were lipemic, icteric or hemolytic specimens, insufficient sample volume and incomplete data.

Sufferers were categorised based on symptoms that have been determined by the Indonesian Ministry of Health 2020. Mild COVID-19 included confirmed COVID-19 cases showing mild and moderate symptoms. Mild symptoms include symptomatic cases without evidence of viral pneumonia or hypoxia. Symptoms include fever, cough, fatigue, anorexia, shortness of breath and myalgia. They may be accompanied by sore throat, nasal congestion, headache, diarrhoea, nausea, vomiting, loss of smell (anosmia), or loss of taste (ageusia). Moderate symptoms include cases with clinical signs of pneumonia (fever, cough, shortness of breath and rapid breathing) without signs of severe pneumonia, including SpO _2_ >93% on room air. Severe COVID-19 included confirmed COVID-19 cases with severe and critical symptoms. Severe symptoms include clinical signs of pneumonia (fever, cough, shortness of breath and rapid breathing) plus either respiratory rate >30 times/minute, severe respiratory distress, or SpO2 <93% on room air. Critical COVID-19 included cases with ARDS, sepsis, or septic shock.

Serum IL-6 levels (pg/mL) were measured by the electrochemiluminescence immunoassay (ECLIA) method using the reagent of Elecsys IL-6 in an automatic Cobas e411 analyzer from Roche diagnostics. The NLR is a comparison in percentage value of total neutrophil and total lymphocyte counts obtained from a complete blood count on Sysmex XS-800i.

Our complete method has been published on protocol.io (DOI: dx.doi.org/10.17504/protocols.io.rm7vzbworvx1/v1). ^17^

### Selection criteria

The inclusion criteria were as follows: patients with COVID-19 confirmed by RT-PCR; the diagnosis was made by a clinician in the Department of Internal Medicine/Pulmonology based on anamnesis and physical, laboratory and radiological examinations; and willingness to participate in the study by providing written informed consent. The exclusion criteria were as follows: lipemic, icteric, or hemolysed specimens, insufficient sample volume and incomplete data.

### Research setting

This study was conducted in several research locations in Makassar, Indonesia: the Outpatient and Inpatient Department/ICU, RSPTN Hasanuddin University (UH RSPTN) Makassar and Makassar City Hospital for sampling, Laboratory of Siloam Hospital Makassar for the IL-6 measurement and Clinical Pathology Laboratory at RSPTN UH for complete haematology examination. The research was conducted in August 2020.

### Research sample

In this study, 3 mL of whole blood placed in ethylenediaminetetraacetic acid (EDTA) tubes was used for NLR examination from complete haematology, and 3 mL of the serum placed in plain tubes was used for IL-6 examination.

### Research design

The research design was an analytic observational study with a cross-sectional approach. Each task in this study was carried out with the permission and knowledge of the patients who provided written informed consent. The study met the ethical requirements of the Health Research Ethics Commission, Faculty of Medicine, Hasanuddin University - Hasanuddin University RSPTN - RSUP Dr. Wahidin Sudirohusodo Makassar (KEPK FKUH-RSPTN UH-RSWS) No. 422/UN4.6.4.5.31/PP36/2020 dated 12 ^th^ August 2020 at Hasanuddin University.

### Research procedure

Subjects consisted of 91 patients diagnosed with COVID-19 were recruited using exhaustive sampling. Namely, the researchers approached all patients suffering from COVID-19 at the Hasanuddin University Hospital and Makassar City Hospital, South Sulawesi, Indonesia. Research subjects who met the inclusion criteria were divided into two groups, namely mild and severe COVID-19. The identity of patients who met the inclusion criteria was recorded and they were provided with a complete explanation of what would be done to them and if they agreed they filled out and signed a written informed consent.

Study subjects who met the inclusion criteria had 3 mL of venous blood taken in an EDTA tube and a normal tube. The blood in the EDTA tube was checked for complete hematology. Serum was obtained after the EDTA tube containing blood formed a clot after 30 minutes at room temperature and was centrifuged for 20 minutes at 3000 rpm. Serum samples were collected until sufficient was obtained and it was stored at -20°C. A minimum of 3 ml of K2-EDTA plasma to prepare for minimum 30 μL of serum is required for the measurement of IL-6. Samples were thawed at 25°C prior to analysis. The following tools and materials were used during the study:

• Cobas e 411 instruments (Cobas e411 analyzer; RRID:SCR_018369)
• IL-6 reagent (Elecsys IL-6; RRID: AB_2938994)
• IL-6 calibrator and control
• Diluents

The test principle used was sandwich with the electrochemiluminescence immunoassay (ECLIA) method. Test work was carried out in advance with a series of preparations including daily calibration and quality control. Sample work was carried out according to the manufacturers’ instructions ( https://diagnostics.roche.com/). The detailed protocol is as follows:

Intended use: Elecsys IL-6 immunoassay is an *in vitro* diagnostic test for the quantitative determination of IL-6 (interleukin-6) in human serum and plasma

Test principle: Sandwich principle.

• First incubation: The sample are at (20-25) ^0^ C before measurement. Then 30 μL of each sample was incubated with 0.9 μg/mL biotinylated monoclonal anti-IL-6-specific mouse antibody (Roche diagnostics, catalogue no. 05109442190).
• Second incubation: After adding a monoclonal IL-6-specific antibody labelled with a ruthenium complex and streptavidin-coated microparticles, the antibodies form a sandwich complex with the antigen of the sample.
• The reaction mixture is aspirated into the measuring cell, where the microparticles are magnetically captured onto the surface of the electrode. Unbound substances are then removed with ProCell/ProCell M/ProCell II M. Application of a voltage of 220V to the electrode induces chemiluminescent emission, measured by a photomultiplier, which is included in Cobas E411instrument.
• Results are determined via a calibration curve which is an instrument- generated explicitly by 2-point calibration, and a master curve provided via the reagent barcode or e-barcode.

Measuring range: 1.5–5000 pg/mL (or 50000 pg/mL if diluted up to 10 times) Reference value: up to 7 pg/mL.

**Elecsys IL-6 reagent pack**

The Elecsys IL-6 immunoassay is an electrochemiluminescence immunoassay “ECLIA” intended for use on cobas e immunoassay analyzers. In this study, we used the Cobas e411 analyzer, which is related to use with the reagent rack pack labeled as IL-6. It consists of:

M Streptavidin-coated microparticles (transparent cap), 1 bottle, 6.5 mL: Streptavidin-coated microparticles 0.72 mg/mL; preservative.

R1 Anti-IL-6-Ab~biotin (gray cap), 1 bottle, 9 mL: Biotinylated monoclonal anti-IL-6 antibody (mouse) 0.9 μg/mL; phosphate buffer 95 mmol/L, pH 7.3; preservative.

R2 Anti-IL-6-Ab~Ru (bpy) (black cap), 1 bottle, 9 mL: Monoclonal anti-IL-6 antibody (mouse) labeled with ruthenium complex 1.5 μg/mL; phosphate buffer 95 mmol/L, pH 7.3; preservative.

**Calibration**

Traceability: This method has been standardized against the NIBSC (National Institute for Biological Standards and Control) 1st IS 89/548 Standard.

Cobas e 411 analyzers: Every Elecsys reagent set has a barcoded label containing specific information for calibration of the particular reagent lot. The predefined master curve is adapted to the analyzer using the relevant CalSet.

The calibration interval may be extended based on acceptable verification of calibration by the laboratory.

### Data analysis

Data were grouped according to the purpose and type, and appropriate statistical methods were selected. Basic data characteristics were described descriptively, and the normality of data was determined by the Kolmogorov–Smirnov test using IBM SPSS statistics version 26 (RRID:SCR_019096). Differences in IL-6 levels and NLR values between the mild and severe COVID-19 groups were analysed using the Mann–Whitney test, and the IL-6 and NLR cut-off values were determined from the receiver operating characteristic (ROC) curve. The correlation between IL-6 and NLR levels and COVID-19 severity was analysed using the chi-square test and the correlation between IL-6 and NLR and COVID-19 was analysed with the Spearman correlation test. The statistical test was significant at p <0.05.

## Results

During the study period, 96 patients with COVID-19 met the inclusion criteria. Five patients were excluded because they did not meet the sample criteria, namely, one patient was lipemic, two involved lysate and two had small serum volumes; thus, they could not be examined. In total, the sample included 91 patients, consisting of 57 (62.6%) with mild COVID-19 and 34 (37.4%) with severe COVID-19. As shown in Table 1, 46 (50.5%) patients were male and 45 (49.5%) were female, and the age range 21–40 years had the highest number of patients. Patients were divided into mild and severe COVID-19, and no significant difference in sex was found among these patients ( Table 2).

**Table 1.** Characteristics of research subjects.

Variable	Amount (%)	COVID-19		p
		Mild (57) n (%)	Severe (34) n (%)	
**Gender**				
Male	46 (50,5)	27 (47,4)	19 (55,9)	0,432 *
Female	45 (49,5)	30 (52,6)	15 (44,1)	
**Age (years)**				
<= 20	3 (3,3)	1 (1,8)	2 (5,9)	0.000 **
21–40	44 (48,4)	38 (66,7)	6 (17,6)	
41–60	24 (26,4)	8 (14)	16 (47)	
61–80	20 (22)	10 (17,5)	10 (29,5)	
**NLR**				
≤ 3,13	47 (51,6)	45 (78,9)	2 (5,9)	0.000 *
> 3.13	44 (48,4)	12 (21,1)	32 (94,1)	

* Chi Square test.

** Kolmogorof Smirnov test.

**Table 2.** Differences in interleukin-6 (IL-6) and neutrophil–lymphocyte ratio (NLR) levels in mild and severe COVID-19.

COVID-19	IL-6 (pg/mL) Median (min-max)	p-value	NLR Median (min-max)	p-value
Mild (n=57)	3,59 (1,50-638,30)	0,000 *	2,18 (0,69-15,58)	0,000 *
Severe (n=34)	28,82 (5,52-926,30)		8,13 (2,24-30,90)	

* Mann Whitney test.

Table 1 shows that in the mild COVID-19 group, the highest number of patients were in the age range of 21–40 years, whereas in the severe COVID-19 group, most of the sufferers were in the age range of ≥41 years. A significant difference was found between the two groups, and this indicates that older age can be a comorbid factor in the development of COVID-19, which becomes more severe.

Based on the NLR cut-off category of 3.13, most of the patients (n = 57 [78.9%]) in the mild COVID-19 group had NLR below the cut-off; in the severe COVID-19 group (n = 34 [94.1%]), the NLR values were above the cut-off (P <0.005). In a 2020 study in China, Yang found that old age and NLR were significantly related to COVID-19 severity. ^18^

In this study, the IL-6 level and NLR were not normally distributed (Kolmogorov–Smirnov); thus, the differences in IL-6 levels and NLR in the two COVID-19 groups were analysed using the Mann–Whitney test. The median serum IL-6 in the mild COVID-19 group was significantly lower (3.59 pg/mL [min–max of 1.50–638.30]) than that of the severe COVID-19 (28.82 pg/mL [min–max of 5.52–926.30], P <0.005). The median NLR in the mild COVID-19 group was significantly lower (2.18 [min–max of 0.69–15.58]) than that of the severe COVID-19 group (8.13 [min–max of 2.24–30.90, P <0.005]).

**Figure 1.**
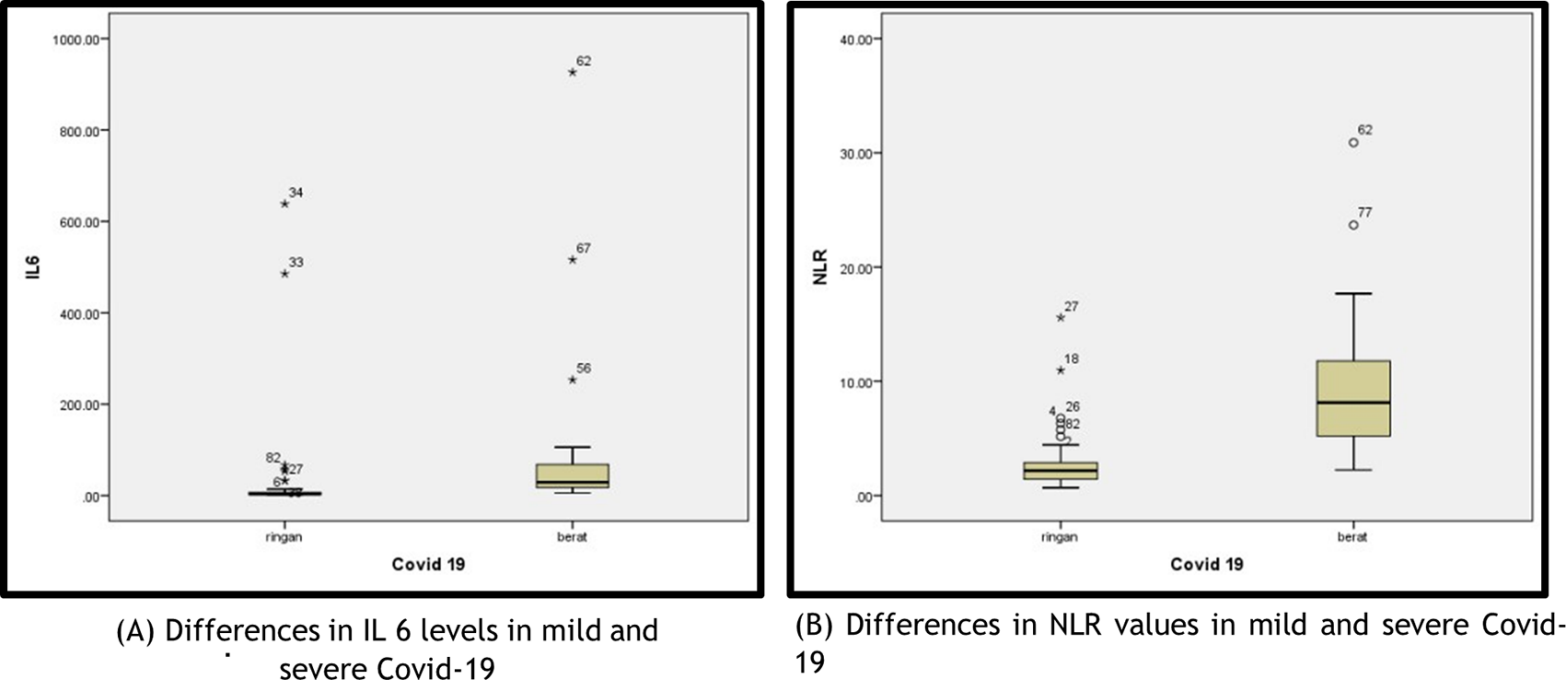
(A) Differences in Interleukin-6 (IL-6) levels in mild and severe COVID-19; (B) Differences in neutrophil–lymphocyte ratio (NLR) values in mild and severe COVID-19.

In Figure 1 in the mild COVID-19 group, two patients had IL-6 levels higher than the average IL-6: a female patient (aged 68 years) who had an IL-6 level of 638.3 pg/mL and NLR of 2.81, who was diagnosed with mild COVID-19 accompanied by diabetes mellitus and hypertension; a male patient (aged 29 years) who had an IL-6 level of 485.4 pg/mL and NLR of 1.26, who was diagnosed with mild COVID-19 accompanied by fever, cough and nausea.

In the severe COVID-19 group, three patients had IL-6 levels higher than the average IL-6: a male patient (aged 73 years) who had an IL-6 level of 926.3 pg/mL and NLR of 30.9 and C-reactive protein (CRP) of 340.2 mg/dL, who was diagnosed with severe COVID-19 accompanied by bilateral pneumonia, cardiomegaly and aortic dilatation; a female patient (aged 60 years) who had an IL-6 level of 515.8 pg/mL and NLR of 2.82, who was diagnosed with severe COVID-19 accompanied by diabetes mellitus and hypertension; and a female patient (aged 54 years) who had an IL-6 level of 105.7 pg/mL, NLR of 17.68 and CRP level of 399.88 mg/dL, who was diagnosed with severe COVID-19 accompanied by hematochezia with suspected malignancy. In the severe COVID-19 group, two patients had an NLR higher than the existing average: a male patient (aged 73 years), with NLR of 30.9, who also had very high IL-6 levels, and was diagnosed with severe COVID-19 accompanied by bilateral pneumonia, cardiomegaly and aortic dilatation and a male patient (aged 58 years) who had an NLR of 23.68 and was diagnosed with severe COVID-19 accompanied by pneumonia and aortic atherosclerosis.

**Figure 2.**
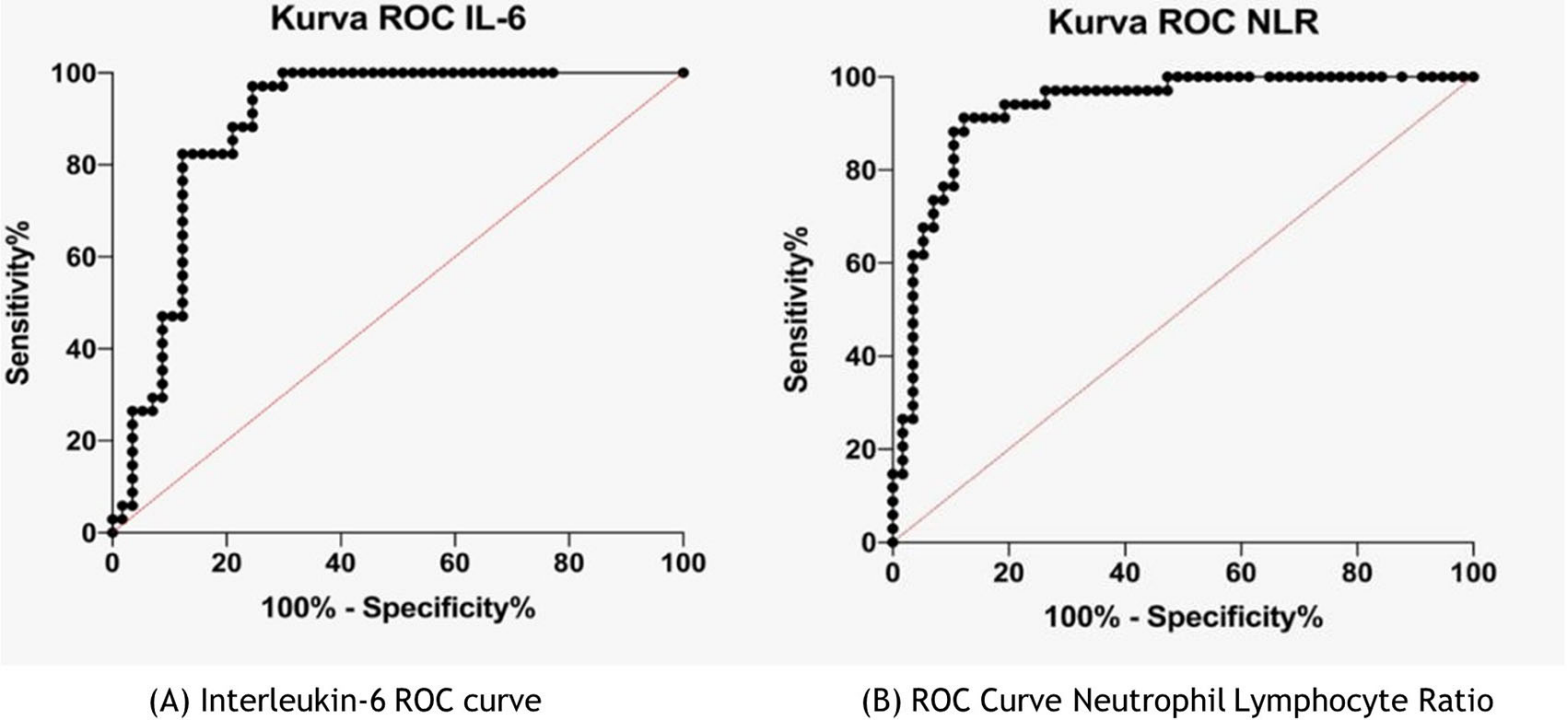
(A) Interleukin-6 receiving operator characteristic (ROC) curve; (B) Neutrophil-lymphocyte ratio ROC curve.

Figure 2 shows the IL-6 and NLR cut-off; the ROC curve was used. In the IL-6 ROC curve, the area under the ROC curve (AUC) was 0.89 (95% CI 0.818–0.957; p <0.0001, and the IL-6 cut-off was >6.99 pg/mL). In the NLR ROC curve, the AUC was 0.93 (95% CI 0.881–0.984; p <0.0001), and the cut-off NLR was >4.18.

The relationship between IL-6 and NLR and COVID-19 severity was assessed using the chi-square test. The results are shown in Tables 3 and 4. As shown in Table 3, more patients with mild COVID-19 (n = 43, 75.44%) had IL-6 levels below the cut-off compared with those (n = 14, 24.56%) who had levels above the cut-off. In the severe COVID-19 group, more patients (n = 33, 97.06%) had IL-6 levels above the cut-off. In the chi-square test, a significant relationship was found between IL-6 levels and COVID-19 severity (P = 0.004; P <0.05). Patients with high IL-6 levels have 29 times the risk of suffering from severe COVID-19.

**Table 3.** Correlation of interleukin-6 (IL-6) levels with the severity of COVID-19.

		Severity of COVID-19		p	OR
		Mild	Severe		
IL-6 levels (pg/mL)	≤ 6.99	43 (75,44%)	1 (2.94%)	0.004 *	29.29
	>6.99	14 (24,56 %)	33 (97,06 %)		

* Chi-square-test.

**Table 4.** Correlation of neutrophil–lymphocyte ratio (NRL) levels with the severity of COVID-19.

		Severity of COVID-19		p	OR
		Mild	Severe		
NRL levels	≤4,18	50 (87,72%)	3 (8,82 %	0.000 *	26.19
	>4.18	7 (12,28 %)	31 (91,18%)		

* Chi-Square test.

The results of the correlation analysis between NLR and COVID-19 are shown in Table 4. In the mild COVID-19 group, more patients (n = 50, 87.72%) had NLR values below the cut-off than those (n = 7, 12.28%) who had levels above the cut-off. In the severe COVID-19 group, 31 (91.18%) patients had NLR values above the cut-off. In the chi-square test, a significant relationship was found between the NLR and the degree of COVID-19 severity (P = 0.000; P <0.05). Patients with a high NLR had 26 times the risk of suffering from severe COVID-19.

**Figure 3.**
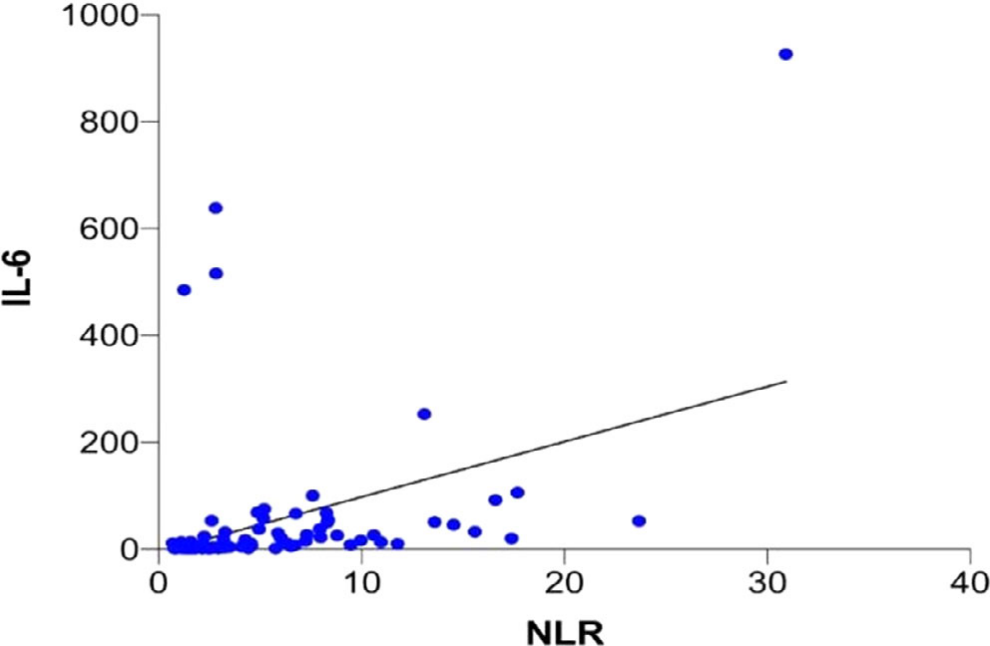
Correlation of Interleukin-6 (IL-6) and neutrophil–lymphocyte ratio (NLR) levels in COVID-19.

The correlation between the serum IL-6 level and the NLR in COVID-19 was analysed using the Spearman correlation test, and the results are shown in Table 5 and Figure 3. After the Spearman correlation test, a P value of <0.001 was obtained, which indicated a significant correlation between the IL-6 level and the NLR in patients with COVID-19. A correlation coefficient of 0.612 (95% CI 0.459–0.729) was obtained indicating a moderate positive correlation. This suggested that an increase in the NLR is associated with an increase in IL-6 levels. However, this relationship may still be influenced by other internal factors. Our raw data has been published on figshare ( https://www.doi.org/10.6084/m9.figshare.22736474). ^19^

**Table 5.** Correlation of serum IL-6 levels with neutrophil–lymphocyte ratio (NLR) values in COVID-19.

	NLR
*Interleukine*-6 serum (pg/mL)	r = 0,612 p <0,001 n = 91

* Spearman test.

## Discussion

The COVID-19 guidelines in Australia and New Zealand, released in March 2020, identified lymphopenia and neutrophilia as prognostic markers of severe COVID-19, and the Centers for Disease Control and Prevention in the United States released guidelines emphasising that leukopenia (9%–25%), leukocytosis (24%–30%) and lymphopenia (63%) are the results of routine blood tests in patients who are hospitalised for COVID-19 ( https://www.who.int/). ^20^

Haematological parameters in patients with COVID-19 demonstrated significant changes including decreased lymphocyte, haemoglobin and platelet counts, whereas combined parameters such as NLR, monocyte–lymphocyte ratio (MLR) and platelet–lymphocyte ratio (PLR) in patients with COVID-19 significantly increased, indicating that changes in routine blood parameters have clinical significance. ^21^^,^^22^ In patients with severe COVID-19 and poor outcomes, the lymphocyte, eosinophil and platelet counts increased, whereas neutrophil, NLR, IL-6, procalcitonin, D-dimer, ferritin, amyloid A protein and CRP levels increased. ^23^^,^^24^

The NLR was identified as a marker of systemic inflammation and a prognostic factor for mortality in patients with COVID-19. An imbalance in neutrophil and lymphocyte counts can indicate severe inflammation leading to complications such as sepsis, MODS and ARDS. ^14^^,^^25^

A high NLR can be caused by the dysregulation of inflammatory cytokine expression and the upregulation of genes involved in the lymphocyte apoptosis pathway caused by SARS-CoV-2 infection. ^26^^,^^27^ A high NLR in patients with severe COVID-19 is characterised by an increase in neutrophil count and a decrease in lymphocyte count. The NLR may reflect an imbalance in the inflammatory and immune response in patients with COVID-19. ^23^^,^^27^ Zhu *et al*. (2020) reported that the AUC for IL-6 was 0.84 (95% CI 0.708–0.962) and NLR was 0.69 (95% CI 0.537–0.842), which were slightly lower than our values, but similarly showed better sensitivity in predicting severe COVID-19.

Haematological parameters in patients with COVID-19 change significantly, including a decrease in lymphocyte, haemoglobin and platelet counts. Meanwhile, combined parameters such as the NLR, MLR and PLR in patients with COVID-19 increased significantly. ^6^^,^^15^ This suggests the clinical significance of changes in routine blood parameters. This is reflected in the results of this study, revealing higher NLR in patients with severe COVID-19.

To determine IL-6 and NLR cut-offs, ROC curves are used ( Figure 2). Very few studies have investigated IL-6 and NLR cut-offs as predictors of COVID-19 severity. Several existing studies have found different IL-6 cut-offs. For example, Harold *et al*. (2020) found that at an IL-6 cut-off of >80 pg/mL, the patient may experience respiratory failure and require a ventilator. Liu *et al*. (2020) reported that an IL-6 cut-off of >34.1 pg/mL indicated severe complications. ^7^^,^^9^ Meanwhile, Liu *et al*. (2020) obtained an NLR cut-off of >4.87 (95% CI 0.659–0.850) with a sensitivity of 56.52% and a specificity of 86.89%, which indicated an 8.5 times probability of experiencing critical/severe COVID-19 compared with a lower NLR. ^28^ The combination of NLR and CRP was recommended as a potential predictor of severe COVID-19. AP *et al*. (2020) obtained an NLR cut-off of >3.3, with a sensitivity of 88% and a specificity of 64%, and Liu *et al* (2020) reported that an NLR of >3.13 and age >50 years indicated severe COVID-19 that required intensive care unit (ICU) admission. ^13^^,^^29^

As shown in Table 3, the chi-square test revealed a significant relationship between IL-6 levels and COVID-19 severity (P = 0.004; P <0.05). Patients with high IL-6 levels have 29 times the risk of suffering from severe COVID-19. ^30^ Zhu *et al*. (2020) analysed several inflammatory markers in mild and severe COVID-19 and assessed the association of these markers with COVID-19 severity. They found that high IL-6 levels, CRP and hypertension were independent risk factors of COVID-19 severity. IL-6 levels are significantly higher in severe than in mild COVID-19 at the first onset and 5–10 days afterward. ^10^ Liu *et al*. (2020) found that IL-6 was greatly increased in severe COVID-19 and was significantly associated with fever, increased CRP, LDH and ferritin. IL-6 was positively related to COVID-19 severity. Thus, IL-6 could be a marker for severe COVID-19. ^12^

Fu *et al*. (2020) compared haematological and inflammatory markers in mild and severe COVID-19 and found that leukocytes (white blood cells), NLR, D-dimer and fibrinogen values were significantly higher in severe COVID-19 than in mild COVID-19. The highest NLR AUC value was obtained among the four parameters, with 0.88, and the value was slightly lower than our results; however, their value similarly showed a strong association between NLR and COVID-19 severity. ^31^

Upon the entry of SARS-CoV-2 into the body, the immune system is activated, which causes an increase in several immune cells and the release of pro-inflammatory cytokines, resulting in an inflammatory response in the area of infection. Subsequently, the inflammation spreads throughout the body. Pro-inflammatory cytokines such as IL-6 are produced, and their levels are indirectly affected by the imbalance of neutrophils and lymphocytes. In severe COVID-19, IL-6 production and NLR values are also higher. Zhu *et al*. (2020) found a positive relationship between IL-6 and NLR (r = 0.428, P <0.001). ^30^

In patients with severe COVID-19 and poor outcomes, the lymphocyte, eosinophil and platelet counts decreased, whereas neutrophil, NLR, IL-6, procalcitonin, D-dimer, ferritin, amyloid A protein and CRP levels increased. The NLR was identified as a marker of systemic inflammation and a prognostic factor for mortality in patients with COVID-19. AP *et al*. (2020) and Liu *et al*. (2020) analysed patients with mild and severe COVID-19 in China and found that high NLR and age were closely related to disease severity and were markers of poor prognosis. Thus, the NLR can be an independent prognostic biomarker in the management of COVID-19. Moreover, Liu *et al*. (2020) set an NLR cut-off of 3.13 and an age of 50 years as risk factors of severe COVID-19 requiring ICU admission.

## Conclusions

Serum IL-6 levels and NLR are higher in severe COVID-19 than in mild COVID-19 cases. Serum IL-6 levels and NLR are positively correlated with COVID-19 severity. If an IL-6 level is more than 6.99 pg/mL, then the patient has a 29 times risk of suffering from COVID-19 and if the NLR level is more than 4.18 pg/mL, then the patient has a 26 times risk. Serum IL-6 levels correlate with NLR in COVID-19.

This study was conducted using a cross-sectional design by collecting data when COVID-19 sufferers were hospitalised, no further observations were made on the course of the patient’s illness and the results of IL-6 and NLR examinations were not monitored. Thus, further research is needed to measure serum IL-6 levels and NLR in patients with COVID-19 using an IL-6 cut-off of >6.99 pg/mL and NLR of >4.18, which can be included in the laboratory parameters for the management of patients with COVID-19.

## Consent

Written informed consent for publication of their clinical details was obtained from the patients.

## Data Availability

Figshare: Underlying data for ‘Correlation of serum interleukin-6 levels and neutrophil-lymphocyte ratio in the severity of COVID-19’, https://www.doi.org/10.6084/m9.figshare.22736474. ^19^ Data are available under the terms of the Creative Commons Attribution 4.0 International license (CC-BY 4.0).
