# Supplementary material for: Correlation of serum interleukin-6 levels and neutrophil-lymphocyte ratio in the severity of COVID-19
Source: F1000Res. 2023 Sep 22;12:1189. [Version 1] doi: 10.12688/f1000research.132157.1 (PMC10719560; doi:10.12688/f1000research.132157.1)
Supplement: Peer review history

# Reviewer report: Reviewer response for version 1

**DOI:** 10.5256/f1000research.145062.r222937

**Published:** 2023-12-13

**Authors:** Satyendra K. Tiwary

**Affiliations:** 1 Institute of Medical Sciences, Banaras Hindu University, Varanasi, Uttar Pradesh, India

                             "Correlation of serum interleukin-6 levels and neutrophil-lymphocyte ratio in the severity of COVID-19" is the research article which was conducted more than 3 years back in August 2020 and after 3 years a lot of spectrum and problem with COVID-19 has changed globally leading to less relevance of the article in current situation globally.

 Although a good correlation of the IL-6 and NLR with severity of the COVID-19 is obvious from this research, there are a lot of inclusions to enrich this article in content and relevance.

  1.Sample size of 91 is quite small considering the volume and epidemic of the disease which may be more value if sample size increased as almost every indoor COVID-19 patient were investigated for IL-6 and NLR almost everywhere.

  2. Only two markers are useful in COVID-19, but inclusion of more biomarkers may be of more scientific message considering more strength in the study.

  3. External Validation of the study is lacking which should be included for the authencity and strength in the study.

  4. Flow chart of the methodology should be included for the better perspectives in the understanding.

  5. Immunoassay images added may enrich the manuscript.

  6. Table 1 and Table 2 should be merged for compact data analysis.

  7. Limitation of the study must be added in the conclusion.

Is the work clearly and accurately presented and does it cite the current literature?

Partly

If applicable, is the statistical analysis and its interpretation appropriate?

Yes

Are all the source data underlying the results available to ensure full reproducibility?

Yes

Is the study design appropriate and is the work technically sound?

Yes

Are the conclusions drawn adequately supported by the results?

Yes

Are sufficient details of methods and analysis provided to allow replication by others?

Partly

Reviewer Expertise:

Surgery, Sepsis, Wound, Oncology,Surgical Gastroenterlogy, Phlebology

I confirm that I have read this submission and believe that I have an appropriate level of expertise to confirm that it is of an acceptable scientific standard, however I have significant reservations, as outlined above.

# Reviewer report: Reviewer response for version 1

**DOI:** 10.5256/f1000research.145062.r222933

**Published:** 2023-12-04

**Authors:** Luis Del Carpio-Orantes

**Affiliations:** 1 Grupo de Estudio para el Diagnóstico y Tratamiento de COVID-19, Sociedad Mexicana de Virología, Veracruz, Veracruz, Mexico; 2 Hospital General de Zona 71, Instituto Mexicano Del Seguro Social, Veracruz, Veracruz, Mexico

After reviewing the document, I appreciated an interesting research effort where it was possible to establish IL-6 levels with INL levels, which is relevant since in many scenarios the measurement of IL-6 or other interleukins was not available, which was extremely serious and made it difficult to follow up on patients, mainly those affected by severe COVID-19 who warranted starting treatments such as tocilizumab or other anti-IL6 drugs. Likewise, in those latitudes with great deficiencies, various indices were used, of which the most significant was the INL, predominating its use over other hematological indices, so this work unites the ideal world where IL-6 levels could be measured and the unreal world where great deficiencies meant that other resources that were easily measurable with cytometry were used. Hematology, so I consider its relevance lies in this point, demonstrating that correlation between both measurements. Given the above, I consider it to be a study worthy of indexing.

 I leave a reference in which various hematological indices have been used (Carpio-Orantes *et al.*, 2020 ^1^).

Is the work clearly and accurately presented and does it cite the current literature?

Yes

If applicable, is the statistical analysis and its interpretation appropriate?

Yes

Are all the source data underlying the results available to ensure full reproducibility?

Yes

Is the study design appropriate and is the work technically sound?

Yes

Are the conclusions drawn adequately supported by the results?

Yes

Are sufficient details of methods and analysis provided to allow replication by others?

Yes

Reviewer Expertise:

Internal Medicine, Infectology, Virology, COVID-19, Long COVID, Arbovirus

I confirm that I have read this submission and believe that I have an appropriate level of expertise to confirm that it is of an acceptable scientific standard.

## References

[1] : Neutrophil-to-lymphocyte ratio, platelet-to-lymphocyte ratio and systemic immune-inflammation index in patients with COVID-19-associated pneumonia. *Gac Med Mex* .2020;156(6) : 10.24875/GMM.M21000480 527-531 10.24875/GMM.M21000480 33877106

# Reviewer report: Reviewer response for version 1

**DOI:** 10.5256/f1000research.145062.r222926

**Published:** 2023-11-29

**Authors:** Mukta Pujani

**Affiliations:** 1 Department of Pathology, ESIC Medical College, Faridabad, Faridabad, Haryana, India

The article is a little late as a lot of literature has been published on various parameters in Covid 19.

 But this article can be improved with several suggestions:

 1. A comparative table maybe drawn on the literature on NLR and IL-6 in Covid-19 from across the world especially South-East Asia. A few articles published early on and latest may be added in the table as mentioned below:

- Pujani *et al*. (2021 ^1^). 
- Sayah *et al.* (2021 ^2^). 
- Pujani *et al.* (2021 ^3^).
- Făgărășan et al. (2023 ^4^).

2. Since CBC forms the basis of the calculated index like NLR, other indices like PLR, MLR may also be compared if deemed fit.

 3. Were NLR and IL-6 co-related with any co-morbidities in Covid-19 patients?

Is the work clearly and accurately presented and does it cite the current literature?

Partly

If applicable, is the statistical analysis and its interpretation appropriate?

Yes

Are all the source data underlying the results available to ensure full reproducibility?

Yes

Is the study design appropriate and is the work technically sound?

Yes

Are the conclusions drawn adequately supported by the results?

Yes

Are sufficient details of methods and analysis provided to allow replication by others?

Yes

Reviewer Expertise:

Immunology, hematology, pathology

I confirm that I have read this submission and believe that I have an appropriate level of expertise to confirm that it is of an acceptable scientific standard, however I have significant reservations, as outlined above.

## References

[1] : An analysis of hematological, coagulation and biochemical markers in COVID-19 disease and their association with clinical severity and mortality: an Indian outlook. *Am J Blood Res* .2021;11(6) :580-591 35103112 PMC8784646

[2] : Interleukin-6, procalcitonin and neutrophil-to-lymphocyte ratio: Potential immune-inflammatory parameters to identify severe and fatal forms of COVID-19. *Cytokine* .2021;141: 10.1016/j.cyto.2021.155428 155428 10.1016/j.cyto.2021.155428 33550165 PMC7834734

[3] : Association of Hematologic biomarkers and their combinations with disease severity and mortality in COVID-19- an Indian perspective. *Am J Blood Res* .2021;11(2) :180-190 34079633 PMC8165721

[4] : IL-6 and Neutrophil/Lymphocyte Ratio as Markers of ICU Admittance in SARS-CoV-2 Patients with Diabetes. *Int J Mol Sci* .2023;24(19) : 10.3390/ijms241914908 10.3390/ijms241914908 37834356 PMC10573809

# Reviewer report: Reviewer response for version 1

**DOI:** 10.5256/f1000research.145062.r209210

**Published:** 2023-09-28

**Authors:** Zhongheng Zhang

**Affiliations:** 1 Department of Emergency Medicine, Sir Run Run Shaw Hospital, Zhejiang University School of Medicine, Hangzhou, China

This is a study comparing the IL-6 and NLR in severe versus mild COVID-19 patients; however, due to the pandemic of COVID-19; the sample size is too small and the simple biomarker to associate severity of COVID-19 lacks novelty. More specifically, I have several comments:

1. IL-6 is a well known biomarker in COVID-19, and this has already been used in clinical practice to guideline immunomodulatory therapy. I wonder what the rationale was to perform the current study. 
2. "no study in Indonesia has reported cut-offs for IL-6 and NLR" - this motivation does not make sense because COVID-19 is a global threat; there is no special traits for Indonesia patients. 
3. IL-6 is a simple marker of the inflammatory system; there are many other biomarkers, and one marker cannot fully reflect the immnue profile. 
4. Table 1 should give full characteristics of the cohorts, many important aspects are missing such as comorbidities, PF ratio, and severity of illness by SOFA.
5. Table 2 is redundant, and can be combined with Table 1. 
6. The lack of external validation prohibits use of the current results to other institutions. 

Is the work clearly and accurately presented and does it cite the current literature?

Partly

If applicable, is the statistical analysis and its interpretation appropriate?

Yes

Are all the source data underlying the results available to ensure full reproducibility?

No

Is the study design appropriate and is the work technically sound?

Yes

Are the conclusions drawn adequately supported by the results?

Yes

Are sufficient details of methods and analysis provided to allow replication by others?

Yes

Reviewer Expertise:

ICU, mortality, sepsis

I confirm that I have read this submission and believe that I have an appropriate level of expertise to state that I do not consider it to be of an acceptable scientific standard, for reasons outlined above.
